# The complete mitochondrial genome of *Cornus officinalis* reveals a multipartite structure and clarifies its phylogenetic position

**DOI:** 10.1080/23802359.2026.2658962

**Published:** 2026-04-20

**Authors:** Mary Tolulope Olutayo, Zhuo Jiang, Hao Zhou, Hengchang Wang, Huajie Zhang

**Affiliations:** a State Key Laboratory of Plant Diversity and Specialty Crops, Wuhan Botanical Garden, Chinese Academy of Sciences, Wuhan, China; b College of Life Science and Technology, Huazhong University of Science and Technology, Wuhan, China

**Keywords:** *Cornus officinalis*, genome structure, mitochondrial genome, phylogeny

## Abstract

*Cornus officinalis* Siebold & Zucc. 1839 is a medicinally important species of Cornaceae, yet mitochondrial genomic information for this genus has remained unavailable. In this study, we assembled and annotated the mitochondrial genome of *C. officinalis* using PacBio HiFi sequencing data. The mitogenome comprises three circular molecules totaling 556,620 bp with similar GC contents of approximately 45%. A total of 70 genes were identified, including 43 protein-coding genes, 23 transfer RNA genes, and four ribosomal RNA genes. Phylogenetic analysis based on mitochondrial protein-coding genes placed *C. officinalis* in a well-supported clade with Hydrangeaceae, Ericaceae, and Primulaceae. This first mitochondrial genome reported for Cornaceae provides a useful resource for mitochondrial genome evolution and angiosperm phylogenetic studies.

## Introduction

The genus *Cornus* Linnaeus (1753), belonging to the family Cornaceae, comprises approximately 55–65 species, most of which are trees and shrubs, with a few perennial herbs (Du et al. 2023a). Species of *Cornus* are mainly distributed in the Northern Hemisphere, with limited representation in tropical Africa and South America (Xiang et al. 2006). Recent molecular phylogenetic studies have showed four main groups in *Cornus*: the cornelian cherries (CC), the big-bracted dogwoods (BB), the dwarf dogwoods (DW), and the blue- or white-fruited dogwoods (BW) (Lindelof et al. 2020). *Cornus officinalis* Siebold & Zucc. 1839 belongs to the cornelian cherry clade and is a shrub or small tree native to East Asia, including China, Korea, and Japan (Xiang et al. 2005; Fang and Hu^1990^). Its height ranges from 4 to 10 m (Dong et al. 2018). Owing to its medicinal value, *C. officinalis* has been extensively studied for its ethnopharmacological and pharmacological properties, including anti-inflammatory and antioxidant activities (Gao et al. 2021).

Although its ethnopharmacology and chloroplast genome have been well documented, mitochondrial genomic information for *C. officinalis*, and for *Cornus* as a whole, has remained unavailable. Recent advances in high-throughput DNA sequencing technologies have enabled the efficient assembly of plant mitochondrial genomes directly from total cellular DNA sequencing data (Ni et al. 2025), providing new opportunities to explore mitogenomic diversity in previously understudied lineages. In this study, we present the first complete assembly and annotation of the *C. officinalis* mitochondrial genome and conduct a mitogenome-based phylogenetic analysis to clarify its evolutionary position, providing new genomic insights into this pharmacologically important species.

## Materials and methods

**Figure 1.**
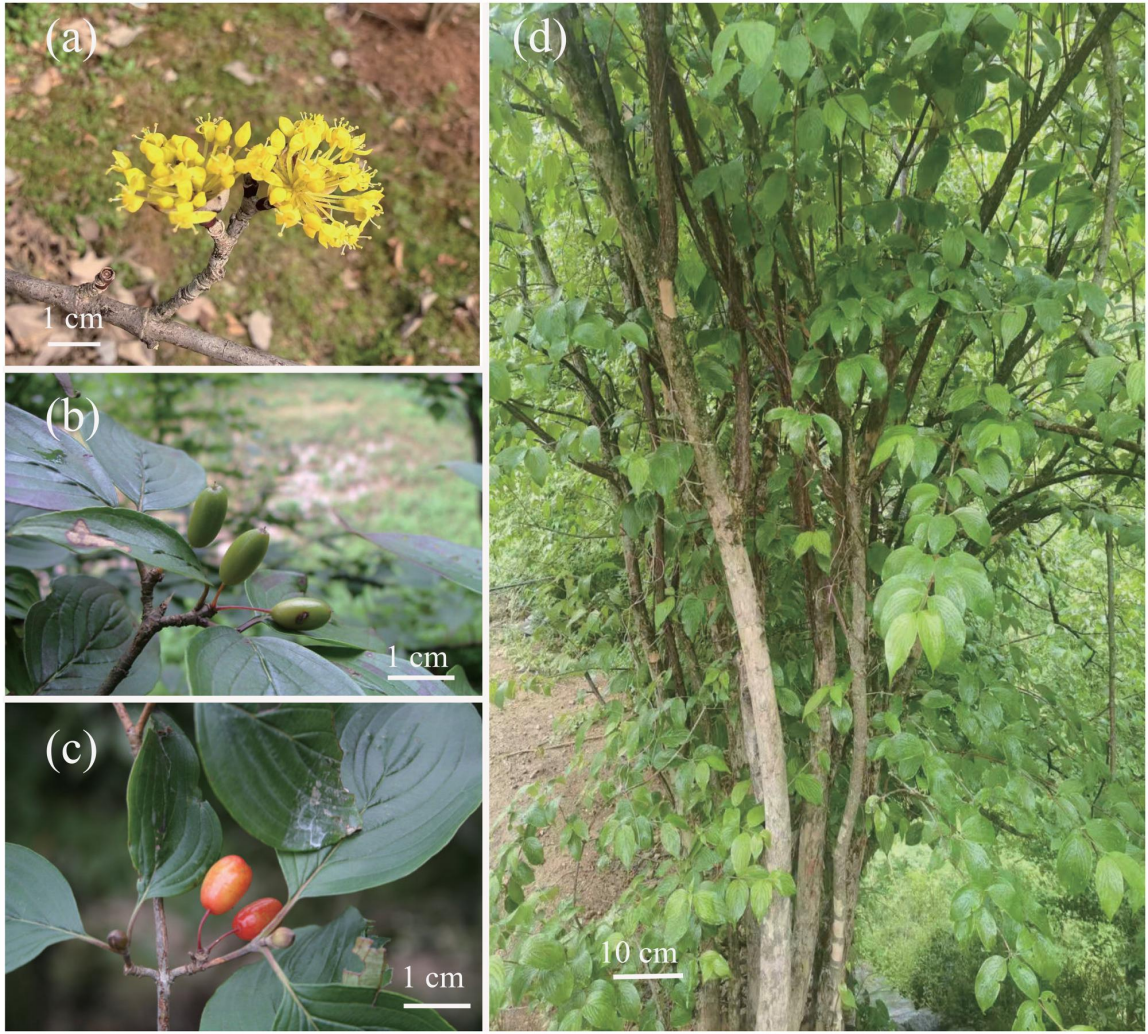
Morphological characteristics of *Cornus officinalis*. (a) Flowers showing typical umbellate inflorescences with yellow petals, which emerge before the leaves. (b) Immature oblong fruits. (c) Mature fruits transitioning to a red color. (d) Whole plant habit, displaying a deciduous tree/shrub form with opposite, ovate-lanceolate leaves. Photographs were taken by Daigui Zhang.

Total genomic DNA was extracted from fresh leaves of *C. officinalis* collected from the Wuhan Botanical Garden (30.5478°N, 114.4155°E) (Figure 1) using the CTAB method (Doyle and Doyle 1987). The collected specimen exhibits typical morphological features of *C. officinalis*: umbellate inflorescences with yellow petals appearing before the leaves (Figure 1a); immature fruits (Figure 1b) that develop into oblong drupes turning red at maturity (Figure 1c); and a deciduous tree/shrub with opposite, ovate-lanceolate leaves (Figure 1d). This specimen was deposited in Herbarium of Wuhan Botanical Garden (HIB), Chinese Academy of Sciences (voucher number: Y. X. Sun 20240501 (HIB); contact: guangwanhu@wbgcas.cn) (Figure S1).

PacBio HiFi sequencing generated approximately 7.6 Gb of clean reads, which were assembled using Flye v2.9.5 with the –meta option enabled (Kolmogorov et al. 2020). Mitochondrial contigs were identified based on mitochondrial gene content. Annotation of the mitochondrial genome of *C. officinalis* was conducted using PMGA (http://47.96.249.172:16084/home) (Li et al. 2025a), followed by manual curation to verify gene boundaries, exon–intron structures, and start/stop codons using Geneious prime (Kearse et al. 2012). The structures of cis-spliced genes were visualized using PMGmap (Zhang et al. 2024).

Phylogenetic analysis was conducted using shared mitochondrial protein-coding genes from twelve angiosperm species representing six families, with *Vitis vinifera* and *Graptopetalum paraguayense* as outgroups (Table S2). Protein coding genes were extracted using PhyloSuite v1.2.3 software (Zhang et al. 2020). A total of 21 genes shared across all sampled individuals were selected for phylogenetic inference, namely *atp1*, *atp4*, *atp6*, *atp8*, *atp9*, *ccmB*, *ccmC*, *ccmFC*, *ccmFN*, *cox1*, *cox2*, *cox3*, *matR*, *nad3*, *nad4*, *nad4l*, *nad6*, *nad7*, *nad9*, *rpl5*, and *rps12*. Sequence alignments were performed using MAFFT v 7 (Katoh and Standley 2013). Poorly aligned regions were trimmed with TrimAl v.1.2 (Capella-Gutiérrez, Silla-Martínez and Gabaldón 2009). The cleaned alignments were then concatenated, the best-fit substitution model was selected using ModelFinder (Kalyaanamoorthy et al. 2017), before the IQ tree was constructed, and a maximum-likelihood phylogenetic tree was constructed with IQ-TREE v2.4.0 (Minh et al. 2020), with node support assessed by bootstrap analysis.

## Results

**Figure 2.**
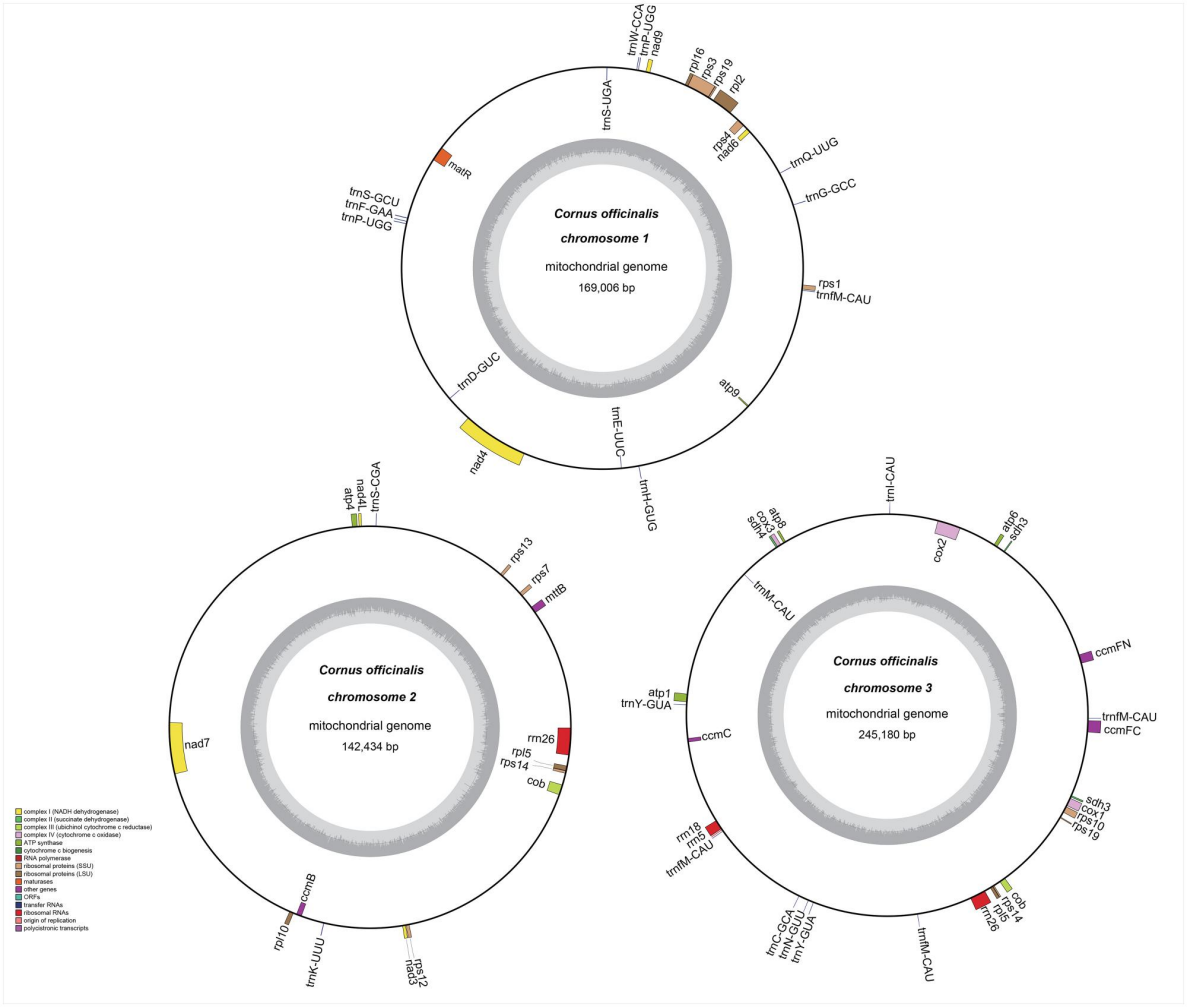
Mitochondrial genome map of *C. officinalis.* Genes located on the outer circle are transcribed clockwise, whereas genes on the inner circle are transcribed counterclockwise. Functional categories of the annotated genes are indicated in the lower left.

The mitochondrial genome of *C. officinalis* exhibits a multipartite organization consisting of three circular molecules (designated chromosome 1 to chromosome 3), with a total length of 556,620 bp (Figures 2 and S2). Chromosome 3 is the largest (245,180 bp), followed by chromosome 1 (169,006 bp) and chromosome 2 (142,434 bp), and all three molecules show similar GC contents ranging from 45.2% to 45.5%. Graphical visualization of the mitochondrial genome assembly and sequencing depth is displayed in Figure S3.

Gene annotation identified 70 functional genes, including 43 protein-coding genes (PCGs), 23 transfer RNA (tRNA) genes, and four ribosomal RNA (rRNA) genes (Figure 2 and Table S1). Specifically, chromosome 1 contained 14 PCGs and 12 tRNA genes; chromosome 2 harbored 14 PCGs, 2 tRNA genes, and 1 rRNA gene; and chromosome 3 included 19 PCGs, 9 tRNA genes, and 3 rRNA genes. Among the protein-coding genes, ten genes contained introns. Of these, seven were cis-spliced (*cox2*, *nad4*, *nad7*, *ccmFC*, *rpl2*, *rps10*, and *rps3*) (Figure S4), while the remaining three (*nad1*, nad2, and *nad5*) were trans-spliced (Figure S5).

**Figure 3.**
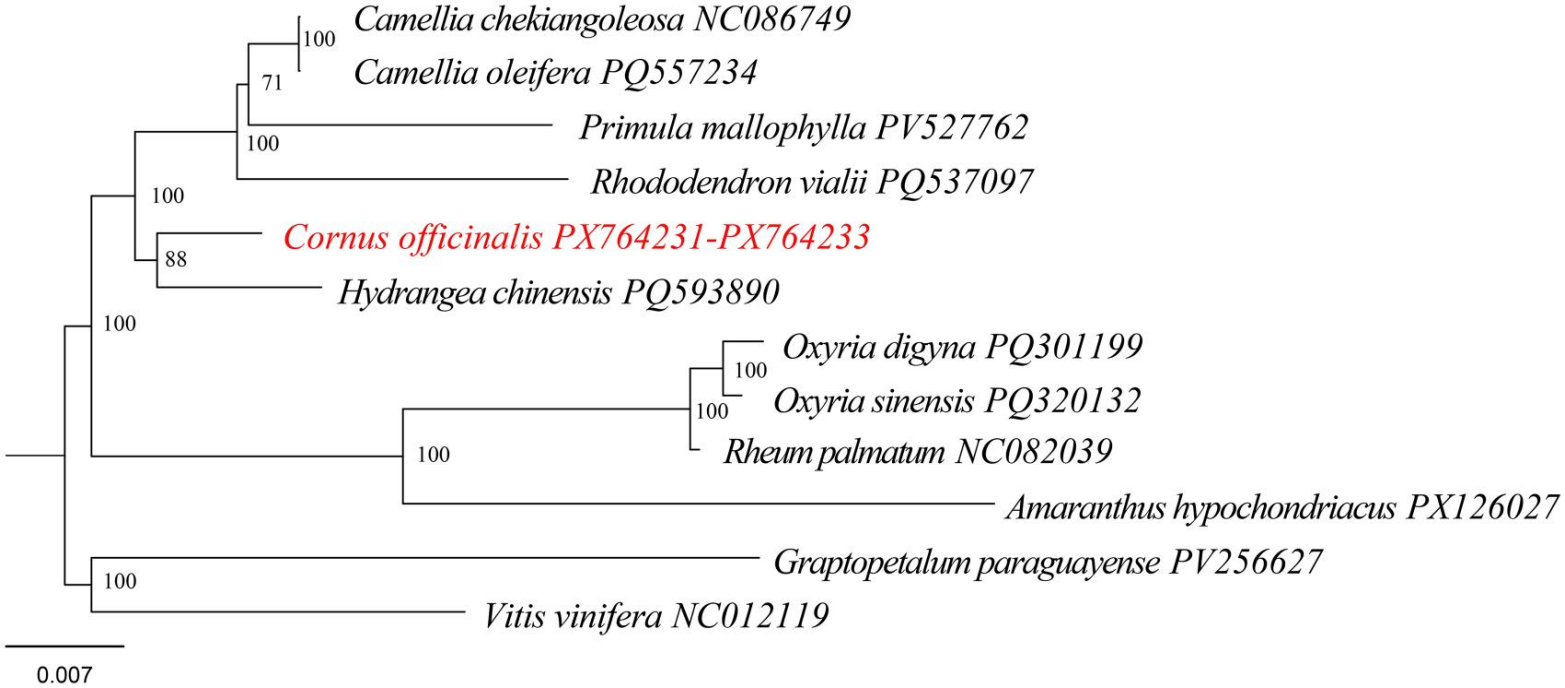
A maximum likelihood tree of *C. officinalis* and related species. Bootstrap support values (based on 1000 replicates) are shown next to the internal nodes. The mitochondrial genome sequences used for phylogenetic analysis included *Camellia chekiangoleosa* (NC_086749.1) (Unpublished), *Camellia oleifera* (PQ557234.1) (Unpublished), *Primula mallophylla* (PV527762.1) (Li et al. 2025b), *Rhododendron vialii* (PQ537097.1) (Lyu et al. 2024), *Hydrangea chinensis* (PQ593890.1) (Unpublished), *Cornus officinalis* (PX764231-PX764233), *Oxyria digyna* (PQ301199.1) (Li et al. 2024), *Oxyria sinensis* (PQ320132.1) (Li et al. 2024), *Rheum palmatum* (NC_082039.1) (Gao et al. 2023), *Amaranthus hypochondriacus* (PX126027.1) (Unpublished), *Graptopetalum paraguayense* (PV256627.1) (Zhou et al. 2025), and *Vitis vinifera* (NC_012119.1) (Goremykin et al. 2009).

Phylogenetic analysis recovered well-resolved relationships among the sampled taxa, with most nodes receiving high bootstrap support (≥88) (Figure 3). *C. officinalis* clustered with *Hydrangea chinensis* and further grouped with representatives of Ericaceae and Primulaceae, whereas Polygonaceae taxa formed a strongly supported monophyletic clade (Figure 3). *Vitis vinifera* and *Graptopetalum paraguayense* were placed at basal positions and served as outgroups (Figure 3).

## Discussion and conclusion

The mitochondrial genome of *C. officinalis* exhibits a typical multipartite architecture comprising three independent circular molecules, all with highly similar GC contents, indicating a broadly homogeneous nucleotide composition across the molecules. In total, 66 functional genes were annotated, including 43 protein-coding genes (PCGs), 23 transfer RNA (tRNA) genes, and four ribosomal RNA (rRNA) genes. However, these genes are unevenly distributed among the three circles. Genes directly involved in oxidative phosphorylation are “partitioned” across different molecules: for example, *atp9* is located on chromosome 1, atp4 on chromosome 2, and *atp1*/*atp6*/*atp8* on chromosome 3. The cytochrome c oxidase genes (*cox1*/*cox2*/*cox3*) are concentrated on chromosome 3, and the complex II–associated genes (*sdh3* and *sdh4*) are also located on chromosome 3, collectively suggesting that the core genetic components supporting the respiratory chain remain intact at the gene-content level. In addition, a relatively complete set of cytochrome c maturation genes is present, with *ccmB* located on chromosome 2 and *ccmC*/*ccmFC*/*ccmFN* on chromosome 3, supporting the essential machinery required for electron transport across the inner mitochondrial membrane.

The exon and intron organization of several genes is notably complex. For instance, *cox2* on chromosome 3 consists of three exons, whereas *rps3* on chromosome 1 contains two exons. Among NADH dehydrogenase–related genes, *nad4* on chromosome 1 is composed of four exons and *nad7* on chromosome 2 comprises five exons (Figure S4). More importantly, the annotation indicates that the five exons of *nad1* are dispersed across chromosomes 1, 2, and 3, while the five exons of *nad2* and *nad5* are distributed between chromosomes 1 and 3 (Figure S5). Such cross-molecule exon distribution implies that the formation of mature transcripts for these genes requires coordinated participation of genomic segments from different circular molecules, highlighting a tight coupling between mitochondrial genome organization and gene expression processes in this species. Furthermore, the presence of duplicated copies of several genes across different molecules is noteworthy. For example, *rrn26* occurs on both chromosomes 2 and 3, and the protein-coding gene *cob* as well as ribosomal protein genes *rpl5*, *rps14*, and *rps19* are also duplicated between chromosomes 2 and 3. These patterns suggest that segmental duplication and genome rearrangement have likely contributed to shaping the present multipartite configuration and gene distribution of the *C. officinalis* mitochondrial genome.

Mitochondrial phylogenetic analyses recovered Cornaceae, Hydrangeaceae, Ericaceae, and Primulaceae as closely related and well-supported clades, consistent with the APG classification framework and previous studies based on chloroplast and nuclear datasets (Group et al. 2016; Fu et al. 2019; Du et al. 2023b). Members of Polygonaceae (Caryophyllales) form a strongly supported monophyletic group and are clearly separated from the asterid-related lineages, in agreement with the placement of Caryophyllales as an independently evolved clade within the core eudicots in APG (Group et al. 2016). Furthermore, Vitaceae and Crassulaceae are positioned toward the basal branches of the phylogenetic tree, consistent with their systematic positions within the APG classification framework and their use as outgroup references in angiosperm phylogenetic analyses (Group et al. 2016).

Notably, this study reports the first complete mitochondrial genome for the family Cornaceae, filling a long-standing gap in mitogenomic data for this lineage. Beyond its phylogenetic significance, the *C. officinalis* mitochondrial genome provides a valuable resource for future investigations into mitochondrial genome evolution, structural variation, RNA editing, and intracellular gene transfer in Cornaceae and related angiosperm lineages.

## Supplementary Material

Supplementary Tables.xlsx

Figure S3.pdf

Figure S1.pdf

Figure S4.pdf

Figure S5.pdf

Figure S2.pdf

## Data Availability

The genome sequence data that support the findings of this study are openly available in GenBank of NCBI under the following accession numbers: chromosome 1 PX764231, chromosome 2 PX764232, and chromosome 3 PX764233. The associated BioProject, SRA, and Bio-Sample numbers are PRJNA1393221, SRR36590266, and SAMN20251224 respectively.
