# Supplementary figures and images for: The complete mitochondrial genome of Cornus officinalis reveals a multipartite structure and clarifies its phylogenetic position
Source: Mitochondrial DNA B Resour. 2026 Apr 20;11(5):659–63. doi: 10.1080/23802359.2026.2658962 (PMC13097176; doi:10.1080/23802359.2026.2658962)

original

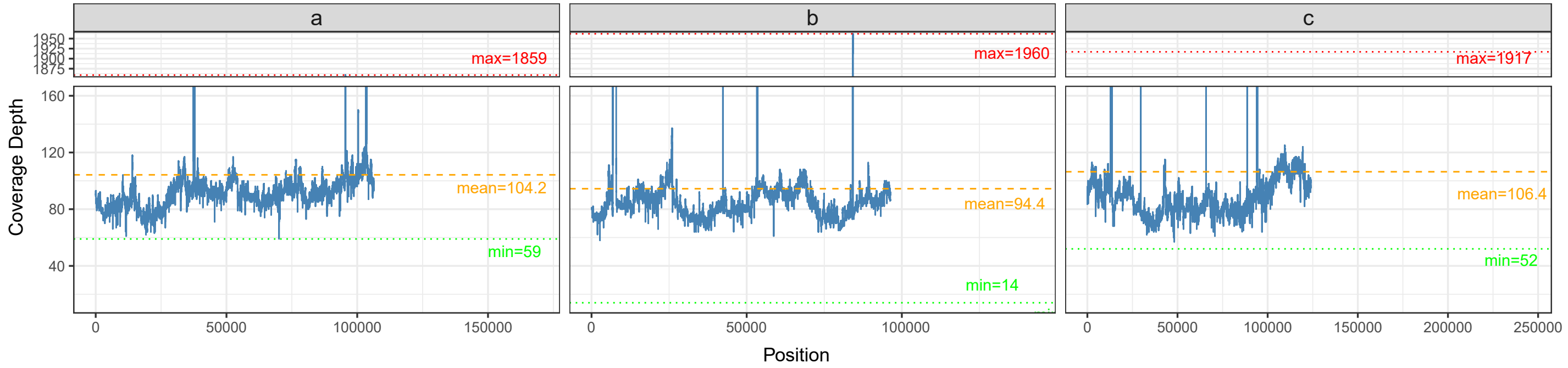

shuffled

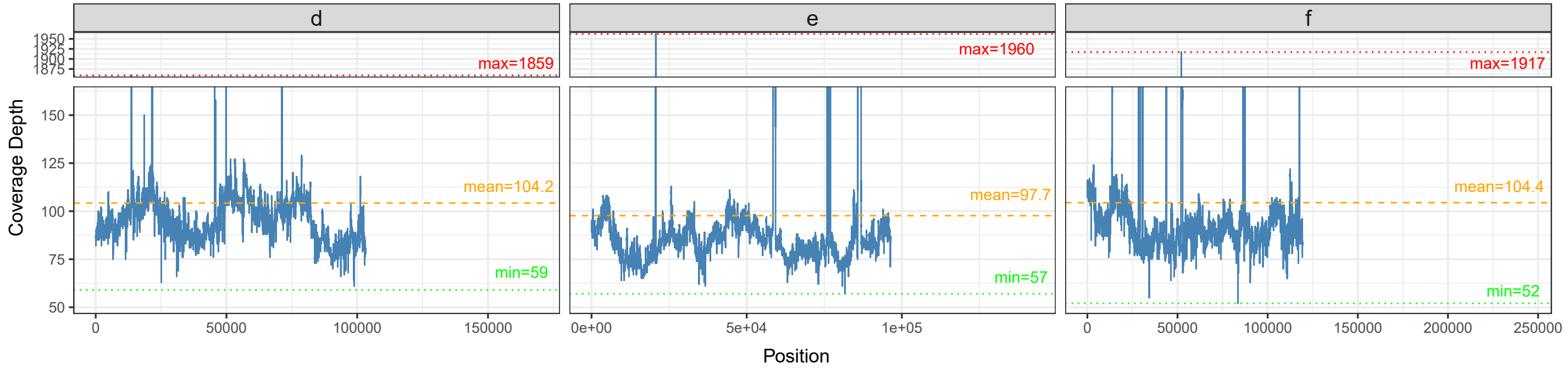

Supplement: Figure S3.pdf [file TMDN_A_2658962_SM0511.pdf]

Y.X. Sun. 20240501

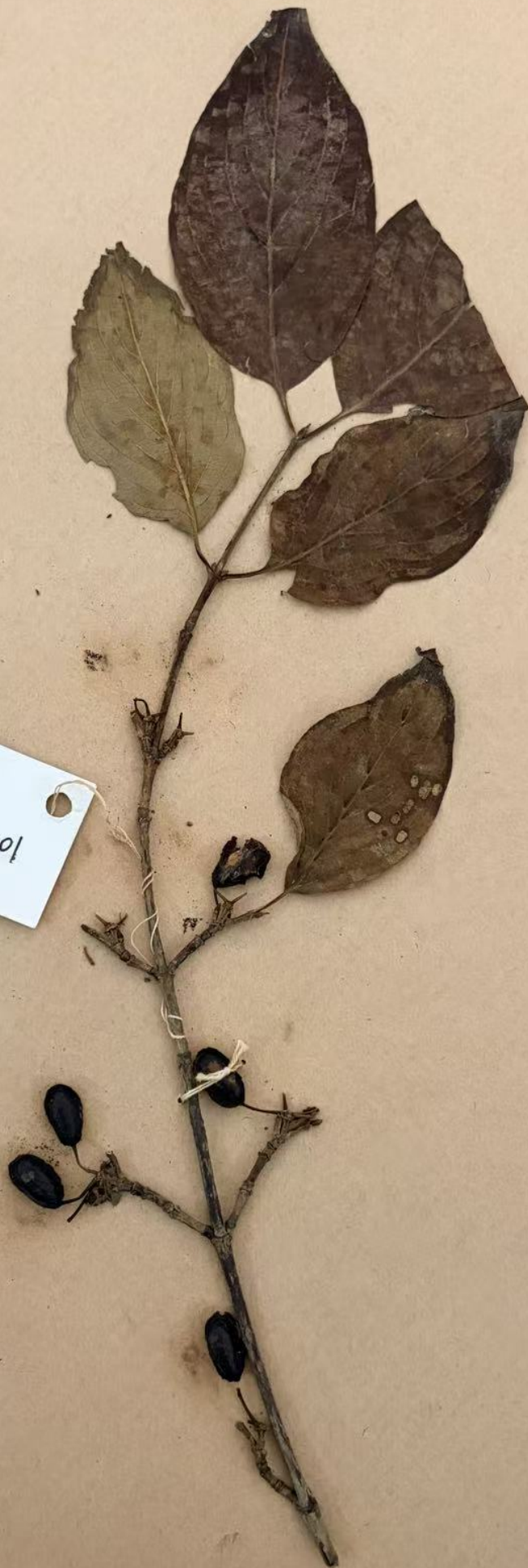

Supplement: Figure S1.pdf [file TMDN_A_2658962_SM0509.pdf]

Schematic of trans-spliced genes in plant mitochondrial genome

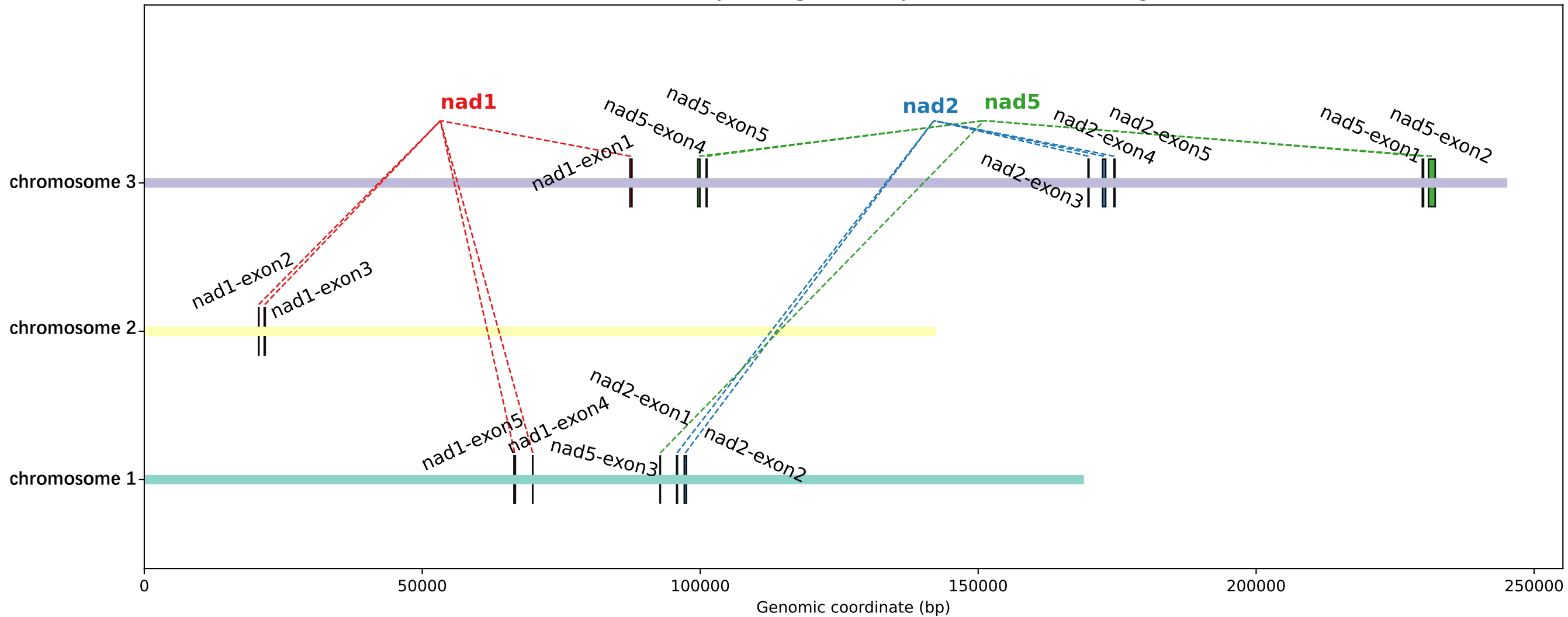

Supplement: Figure S5.pdf [file TMDN_A_2658962_SM0507.pdf]

(a)

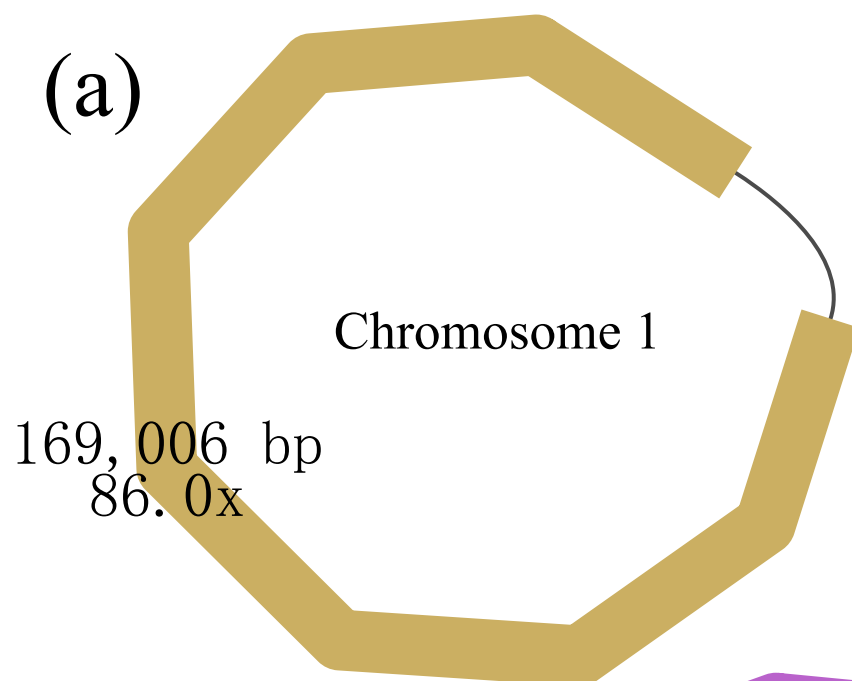

(b)

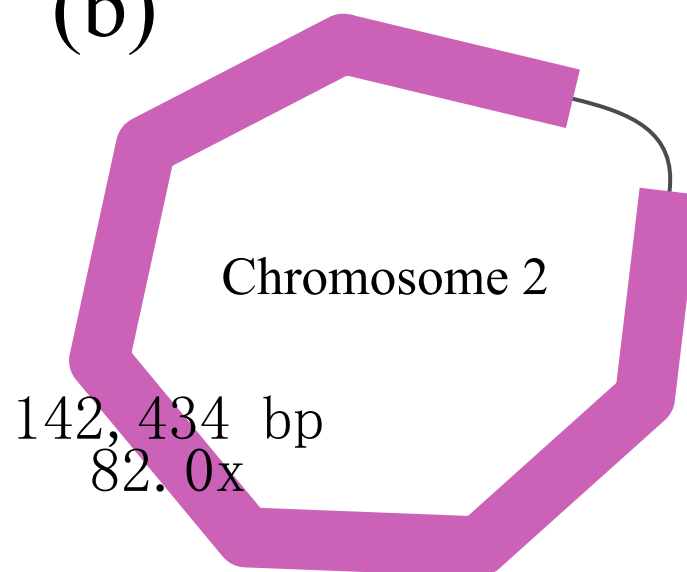

(c)

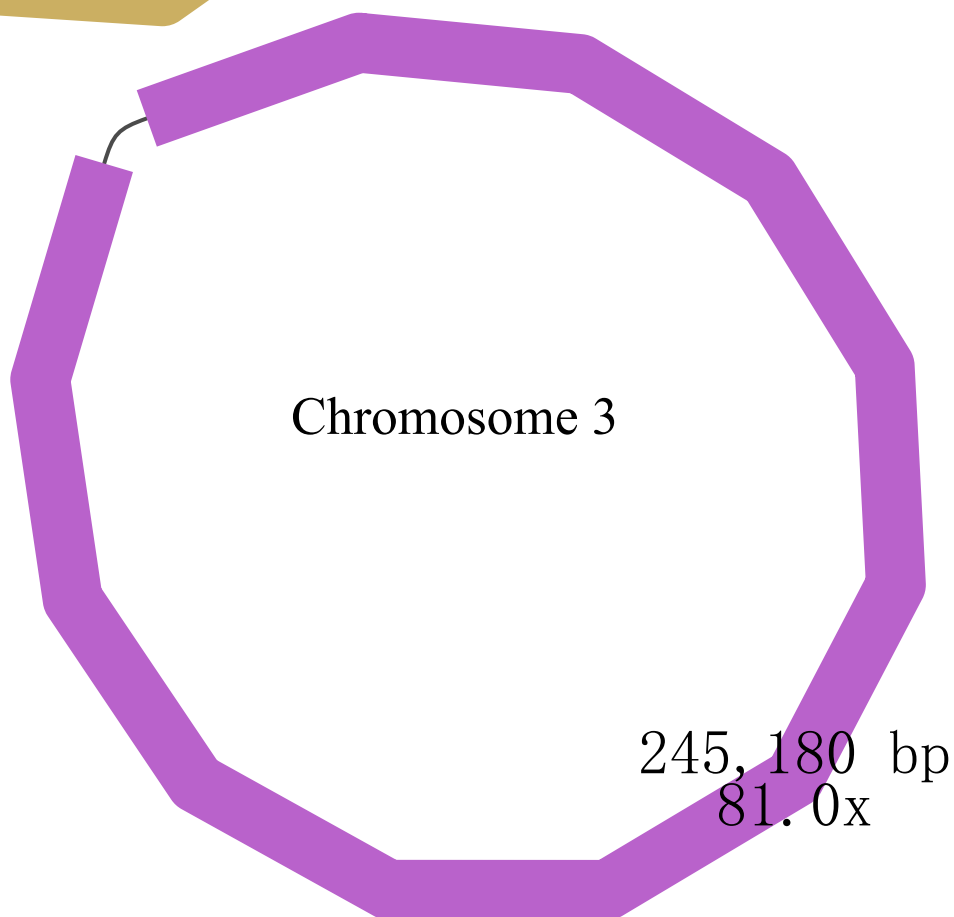

Supplement: Figure S2.pdf [file TMDN_A_2658962_SM0505.pdf]
