# Supplementary material for: The complete mitochondrial genome of Cornus officinalis reveals a multipartite structure and clarifies its phylogenetic position
Source: Mitochondrial DNA B Resour. 2026 Apr 20;11(5):659–63. doi: 10.1080/23802359.2026.2658962 (PMC13097176; doi:10.1080/23802359.2026.2658962)

Cornus officinalis

Mitochondrial Genome
cis-splicing genes

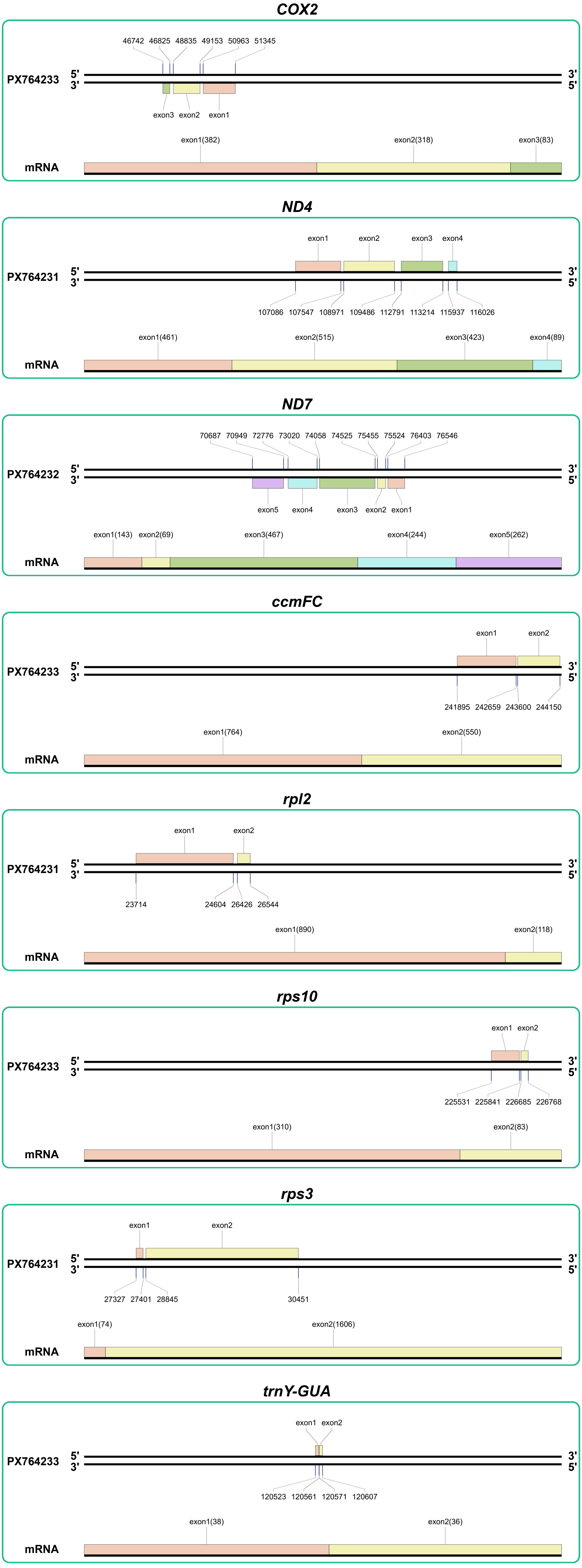

Supplement: Figure S4.pdf [file TMDN_A_2658962_SM0508.pdf]
